# An update on transcriptional and post-translational regulation of brain voltage-gated sodium channels

**DOI:** 10.1007/s00726-015-2122-y

**Published:** 2015-10-27

**Authors:** Donatus O. Onwuli, Pedro Beltran-Alvarez

**Affiliations:** School of Biological, Biomedical and Environmental Sciences, University of Hull, Hardy Building Cottingham Road, Hull, HU6 7RX UK

**Keywords:** Voltage-gated sodium channel, Regulation, Transcription factor, Alternative splicing, Post-translational modification

## Abstract

Voltage-gated sodium channels are essential proteins in brain physiology, as they generate the sodium currents that initiate neuronal action potentials. Voltage-gated sodium channels expression, localisation and function are regulated by a range of transcriptional and post-translational mechanisms. Here, we review our understanding of regulation of brain voltage-gated sodium channels, in particular SCN1A (Na_V_1.1), SCN2A (Na_V_1.2), SCN3A (Na_V_1.3) and SCN8A (Na_V_1.6), by transcription factors, by alternative splicing, and by post-translational modifications. Our focus is strongly centred on recent research lines, and newly generated knowledge.

## Introduction

Voltage-gated sodium channels are essential proteins in brain physiology. Upon voltage-mediated activation, sodium channels produce sodium currents responsible for depolarisation of excitable cells, including neurons and cardiomyocytes. From the point of view of biomedical sciences and pathophysiology, brain disorders such as some forms of epilepsy have long been directly associated with voltage-gated sodium channel malfunction.

Sodium channels are thought to be macromolecular complexes composed of tens of different proteins (Abriel et al. 2015). The pore-forming protein is known as the α subunit, and is sufficient to generate sodium currents. All α subunits include a voltage sensor that promotes channel opening when the cell membrane is depolarized by a few millivolts. Sodium channels thus activate, generate the sodium currents that underlie the initial depolarisation phase of the action potential, and then inactivate within tens of milliseconds, critically shaping cell repolarisation (Zilberter et al. 1994).

There are nine isoforms of the voltage-gated sodium channel α subunit, and each form has distinct expression and electrophysiological patterns. In this review, we have considered the main sodium channel isoforms expressed in the central neuronal system (CNS), i.e., SCN1A (Na_V_1.1), SCN2A (Na_V_1.2), SCN3A (Na_V_1.3) and SCN8A (Na_V_1.6). Wherever relevant we have also included additional information regarding other isoforms, including SCN5A (generally known as the cardiac isoform, Na_V_1.5) and SCN9A (mainly expressed in the peripheral nervous system, Na_V_1.7), (Dib-Hajj et al. 2013).

Sodium channel α subunits are large (ca. 2000 residues), hydrophobic, integral membrane proteins that have been fascinating (and challenging) a range of scientific communities including biochemists, pharmacists, neuroscientists, and electrophysiologists for more than three decades (Catterall 2015). Although detailed mammalian voltage-gated sodium channel structures are not yet available, it is widely accepted that the topology of α subunits at the protein level consists of four homologous domains (termed DI to DIV), each consisting of six transmembrane helices, and joined by cytosolic interdomain linkers (Yu and Catterall 2003). The *N* and *C* termini of α subunits are also intracellular. Thus, cytosolic interdomain linkers, and *N*- and *C*-terminal domains of α subunits are accessible to intracellular enzymes that catalyse post-translational modifications (PTM) of the channels.

In this review, we aim to integrate progress in our understanding of CNS voltage-gated sodium channel regulation at the transcriptional and post-translational level. The reader will find that much more is known on sodium channel PTMs than on the transcriptional mechanisms that regulate channel expression. Consequently, the weight of the review is balanced towards PTMs. Our focus is strongly centred on recent research lines, and newly generated knowledge. The goal is to facilitate dissemination of recent developments with a view on fostering further relevant research.

## Regulation of brain sodium channel expression at the transcriptional level

In this section, we have considered the regulation of CNS voltage-gated sodium channels by transcription factors, and by alternative splicing. The regulation of sodium channels at the post-transcriptional level (e.g., by microRNAs) is out of the scope of the present review.

### Regulation by transcription factors

Promoter regions of brain voltage-gated sodium channel genes have been described, including SCN1A (Dong et al. 2014; Long et al. 2008); SCN2A (Lu et al. 1998; Schade and Brown 2000), SCN3A (Martin et al. 2007), and SCN8A (Drews et al. 2005, 2007). Based on the sequence analyses and databases, several transcription factors have been proposed to control brain sodium channel expression (Long et al. 2008). Experimentally, a recent study has shown that SCN3A expression is regulated by promoter CpG methylation and Methyl-CpG-binding domain protein 2 (MBD2), (Li et al. 2015). MBD2 targets methylated CpG for demethylation, possibly leading to activated transcription. Consistently, knock-down of MBD2 decreased SCN3A mRNA levels in a neuroblastoma cell line. In seizure-induced mice, MBD2 expression was increased, which correlated with decreased CpG methylation, and enhanced SCN3A expression (Li et al. 2015).

Another recent development has been the identification of receptor for activated C kinase 1 (RACK1) as a repressor of SCN1A expression (Dong et al. 2014). The authors identified a transcriptional silencer in a region between +53 and +62 bp downstream of SCN1A promotor and used EMSA assays to uncover possible transcriptional regulators. RACK1 was found to bind to the silencer in NT2 cells (a pluripotent embryonal carcinoma cell line often used for differentiation into neurons). Knocking-down RACK1 in NT2 cells markedly increased SCN1A mRNA levels (Dong et al. 2014).

Sodium channel macromolecular complexes may incorporate proteins classically known as voltage-gated sodium channel β subunits. These include five different proteins termed β1, β1b, β2, β3, and β4. Many groups have studied the effect of β subunits on α subunit trafficking and electrophysiology, mainly from the point of view of protein–protein interactions (for a recent review, see Namadurai et al. 2015).

Additionally, sodium channel β subunits have been proposed to regulate α subunits at the transcriptional level. One of the first experimental observations was the increase in Na_V_1.1 mRNA and protein levels in the presence of proteases targeting the β2 subunit. The group of Kovacs, and others, has demonstrated the sequential mechanism by which, first, ADAM10 and BACE1 proteases cleave off the extracellular domain of the β2 subunit. Second, γ-secretase releases the β2 intracellular domain. And third, the β2 intracellular domain induces an increase in Na_V_1.1 mRNA and protein levels (Kim et al. 2005, 2007; Wong et al. 2005), although the precise pathways for β2 internalisation into the cell nucleus remain unknown. BACE1-dependent sodium channel expression seems to be specific for Na_V_1.1, and mRNA levels of other brain Na_V_ isoforms including Na_V_1.2, Na_V_1.3 and Na_V_1.6 are relatively insensitive to BACE1 protease activity (Kim et al. 2007, 2011).

Likewise β2, the β1 subunit has been shown to regulate Na_V_ expression, and mouse models show changes in brain Na_V_ expression and localization upon β1 deletion (Chen et al. 2004). In a recent development, β1 subunit silencing has been shown to result in decreased Na_V_1.1, Na_V_1.3 and Na_V_1.6 (but not Na_V_1.2) mRNA and protein levels in cells models (Baroni et al. 2014), although the mechanism underlying this regulation was not investigated. Although β1 subunit is a target for BACE1 in vitro, the question remains whether this is physiologically relevant (Wong et al. 2005).

### Regulation by alternative splicing

The first evidences for alternative splicing of brain sodium channels were reported more than 20 years ago (Sarao et al. 1991; Gustafson et al. 1993), and splicing mechanisms are thought to be common to most brain Na_V_ isoforms (Copley 2004). In particular, SCN1A alternative splicing has been extensively studied due to its relevance in CNS disorders such as epilepsy (Lossin 2009; Schlachter et al. 2009; Le Gal et al. 2011; Thompson et al. 2011).

The best studied SCN1A splicing variants are often referred to as the adult and neonatal forms, although both forms are expressed in adults. They result from the mutually exclusive expression of either exon 5A (adult) or 5N (neonatal). Common SCN1A polymorphisms can have a massive effect on the expression of the 5N variant in normal adults (Tate et al. 2005; Heinzen et al. 2007). The 5A/5N splicing event can also be modulated by splice-modifier proteins, including sodium channel modifier 1 (SCNM1). Very recently, a mutation in SCNM1 has been linked to epilepsies possibly via regulation of SCN1A splicing leading to reduction of the variant containing exon 5N (Kasteleijn-Nolst Trenité et al. 2015). SCNM1, as well as other splicing regulators such as Rbfox2, can also modulate SCN8A splicing (Buchner et al. 2003; Gehman et al. 2012).

## Regulation of brain sodium channels at the post-translational level

From biochemical assays in vitro to targeted purification of proteins from tissues, research in sodium channel PTMs has recently expanded from (immuno) chemical methods to embrace mass spectrometry and proteomics. Here, we review our current understanding of some of the best known sodium channel PTMs. As before, we have included Na_V_1.1, Na_V_1.2, Na_V_1.3, and Na_V_1.6. Where relevant, Na_V_1.5 has also been considered because of the wealth of available Na_V_1.5 PTM data. In particular, Na_V_1.5 phosphorylation, ubiquitylation, and arginine methylation have been studied in detail (“Phosphorylation”, “Ubiquitylation” and “Arginine methylation”, respectively).

Previously in “Regulation by transcription factors”, we have reviewed our knowledge of sodium channel β subunit processing by proteases, leading to transcriptional regulation of α subunits. In “Regulation of brain sodium channels by proteases”, we have included available data on direct proteolysis of α subunits. Although proteolysis is in most cases associated with degradation, it can also be regarded as PTM if it is limited and specific (Rogers and Overall 2013).

### Phosphorylation

Phosphorylation is the most experimentally observed PTM at the proteome-wide level, and it is certainly thought to be the most abundant PTM along with *N*-glycosylation (Khoury et al. 2011). Sodium channels are no exception to the rule and phosphorylation is the most studied and observed sodium channel PTM.

#### Identified phosphorylation sites

The aim of this subsection is to comprehensively collect and update the repertoire of sodium channel ‘phosphorylatable’ sites (Table 1). These include phosphosites identified by the use of in vitro assays and heterologous expression experiments, as well as those identified in sodium channels isolated from native sources. Data in Table 1 are taken from classical papers (Berendt et al. 2010), and previous reviews (Cerda et al. 2011; Baek et al. 2011), and updated to include recent original articles that described novel phosphosites (Marionneau et al. 2012; Baek et al. 2014; Herren et al. 2015). Functional consequences of Na_V_ phosphorylation are discussed below.

**Table 1:**
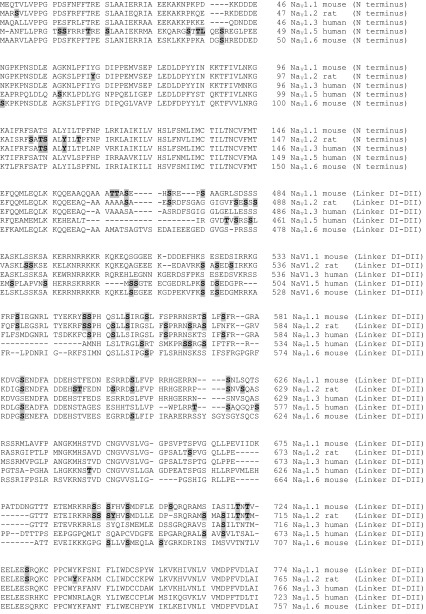

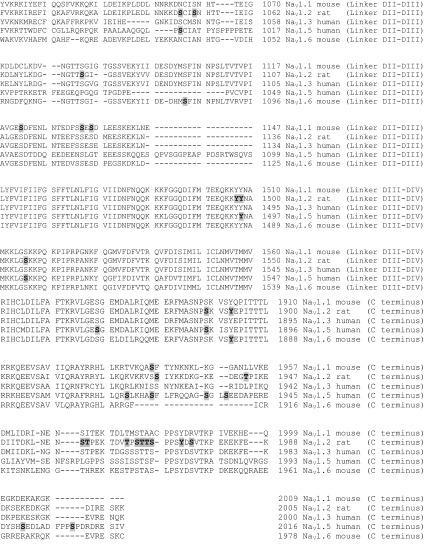
Phosphosites of different Na_V_ isoforms

Phosphorylated residues are shown bold and shadowed

Is it safe to assume that if a Na_V_ isoform is phosphorylated at a certain residue, then our favourite isoform will also be, provided the site is conserved? The general answer is No. Visual analysis of phosphosite conservation in Table 1 leaves little room for hope, at least according to the data currently available. The exception is the interdomain linker between domains I and II, which is considered the PTM hot-spot (Cantrell and Catterall 2001), and where one can find phosphosites conserved among 3, sometimes 4, of the considered Na_V_ isoforms. Nevertheless, due to the substoichiometric and labile nature of phosphorylation, the failure to detect a protein modification does not imply that a residue is not phosphorylated. Perhaps future comprehensive proteomic studies will demonstrate higher degree in phosphosite conservation among Na_V_ isoforms.

#### Specificity of the functional effect of phosphorylation among Na_V_ isoforms

Most of the phosphosites included in Table 1 were identified by proteomics and mass spectrometry methods, and we currently lack information on which protein kinase may catalyse phosphorylation of many of the Na_V_ phosphosites. Nevertheless, it has long been known that protein kinase C (PKC) and cAMP-dependent kinase (PKA) can phosphorylate brain Na_V_ channels (West et al. 1991; Numann et al. 1991; Li et al. 1992, 1993). Other kinases involved in regulating brain Na_V_ phosphorylation are glycogen synthase kinase 3 (GSK3) (James et al. 2015), protein kinase CK2 (Hien et al. 2014), A kinase-anchoring protein 15 (Few et al. 2007), Fyn tyrosine kinase (Beacham et al. 2007), and p38 mitogen-kinase activated protein kinase (Wittmack et al. 2005).

The functional effects of channel phosphorylation on Na_V_ electrophysiology often depend on the specific isoform of interest. For instance, phosphorylation by PKA and PKC results in attenuation of Na_V_1.2 currents due to defective channel trafficking to the cell surface (Li et al. 1992). But Na_V_1.6 channels are relatively insensitive to PKA/PKC regulation (Chen et al. 2008), and Na_V_1.5 currents are enhanced by PKA activation due to increased Na_V_1.5 expression at the cell surface (Hallaq et al. 2006). Subtle variations in the primary sequence of Na_V_ isoforms must underlie such differences. For instance, it is thought that Na_V_1.5 phosphorylation by PKA at S528 masks an endoplasmic reticulum retention signal (RRR_535_), thereby promoting Na_V_1.5 trafficking to the membrane (Zhou et al. 2002). This endoplasmic reticulum signal is absent in Na_V_1.2 (RVK_585_) and modified in Na_V_1.6 (RFR_575_).

Opposite functional effects of post-translational modifications on distinct Na_V_ isoforms have also been observed after phosphorylation by Fyn kinase. Fyn kinase phosphorylates essential tyrosine residues within the inactivation gate of sodium channels, including the equivalent Y1498 (Na_V_1.2) and Y1495 (Na_V_1.5). Yet, the functional effect of phosphorylation by Fyn on channel inactivation is a negative (Na_V_1.2) or positive (Na_V_1.5) shift in the voltage dependence of inactivation (Beacham et al. 2007; Ahern et al. 2005). The simplest explanation is that Fyn phosphorylates other Tyr residues within Na_V_1.2 and Na_V_1.5 sequences, and this has indeed been demonstrated for Na_V_1.2 (including Y66, Y1497, and Y1893), (Beacham et al. 2007). Nevertheless, recent work has reported that distinct splicing variants of the same Na_V_ isoform show different electrophysiological behaviour upon phosphorylation by Fyn, which introduces another level of complexity (Iqbal et al. 2015).

### Ubiquitylation

Protein ubiquitylation (or ubiquitination) is a post-translational modification that involves the orchestrated function of three types of enzymes. First, ubiquitin activating enzyme (E1) catalyses thioester formation between the *C* terminus of ubiquitin and an internal cysteine. Second, activated ubiquitin is transferred to the ubiquitin conjugating enzyme (E2). Third, ubiquitylation of the substrate protein is catalysed by ubiquitin ligases (E3), which covalently attach ubiquitin molecules to lysine residues within the target sequence. Ubiquitylation is often associated with protein degradation.

There are hundreds of E3 ubiquitin ligases, usually classified into two groups: HECT (homologous to E6-AP C terminus) ligases, and RING (really interesting new gene) ligases (Goel et al. 2015). Until 2015, it was thought that only HECT ligases could catalyse sodium channel ubiquitylation (see below).

The most studied molecular mechanism for sodium channel ubiquitylation involves channel recognition by Nedd4-2 ubiquitin ligases (HECT-type ligases) via protein–protein interaction between the WW4 domain of Nedd4-2, and the PY motif of neuronal and cardiac sodium channels (Fotia et al. 2004; van Bemmelen et al. 2004). Ubiquitylation by Nedd4-2 has been shown to tag sodium channels for internalisation from the cell surface, including Na_V_1.2 (Fotia et al. 2004), Na_V_1.6 (Gasser et al. 2010), and Na_V_1.5 (Rougier et al. 2005). However, in most cases, the precise modification site(s), i.e., the Lys residues that are ubiquitylated, remain to be confirmed.

Very recently, compelling evidence has been presented that shows ubiquitylation of sodium channels in zebra fish CNS by RNF121, a member of the RING family of E3 ubiquitin ligases (Ogino et al. 2015). From the initial observation that zebra fish bearing mutations in RNF121 present defective Na_V_ trafficking in neurons and skeletal muscle, the investigators moved on to perform heterologous expression of Na_V_1.6 and RNF121 in HEK 293T cells. Results showed increased Na_V_1.6 degradation upon co-expression of RNF121 but, intriguingly, enhanced Na_V_1.6 membrane localization when co-expressed with RNF121 *and* auxiliary NaV β subunits (Ogino et al. 2015).

### Arginine methylation

Arginine methylation consists on the addition of methyl groups to arginine residues of proteins. Arginine methylation is catalysed by protein arginine methyl transferases (PRMTs) that transfer a methyl group from *S*-adenosyl-*l*-methionine (SAM) to the target arginine. Arginine methylation has recently been reported as a novel post-translational modification of the voltage-gated sodium channel family using Na_V_1.5 as a model system (Beltran-Alvarez et al. 2011).

The groups of Comb and Trimmer have described arginine methylation of brain sodium channels. Using a proteomic approach and bespoke antibodies that recognise peptides bearing methylated arginine, the group of Comb reported arginine methylation of Na_V_1.1, Na_V_1.2 and Na_V_1.5 in the mouse brain (Guo et al. 2014). In parallel, the group of Trimmer described arginine methylation of Na_V_1.2 purified from rat brain (Baek et al. 2014). We analysed the methylation sites reported by the three referenced articles (Beltran-Alvarez et al. 2011; Guo et al. 2014; Baek et al. 2014), and found that three sites have been observed by at least two independent studies (Table 2).

**Table 2:** Hot spots of arginine methylation sites

Methylated residue (rat Na_V_1.2 numbering)	Isoform where methylation was observed (isoform numbering)	Species and tissue	References	Notes
R563	Na_V_1.1 (560)	Mouse, brain	Guo et al. 2014	Observed in human Na_V_1.5 (R513) expressed in HEK 293 cells (Beltran-Alvarez et al. 2011). Na_V_1.5 peptide containing R513 is methylated in vitro by PRMT3 (Beltran-Alvarez et al. 2015).
	Na_V_1.2 (563)	Mouse, brain	Guo et al. 2014	
	Na_V_1.2 (563)	Rat, brain	Baek et al. 2014	
R570	Na_V_1.2 (570)	Mouse, brain	Guo et al. 2014	
	Na_V_1.2 (570)	Rat, brain	Baek et al. 2014	
R574	Na_V_1.5 (526)	Mouse, brain	Guo et al. 2014	Observed in human Na_V_1.5 (R526) expressed in HEK 293T cells (Beltran-Alvarez et al. 2011).
	Na_V_1.5 (526)	Heart, human	Beltran-Alvarez et al. 2014	

Methylation sites reported by at least two independent studies

The functional consequences of sodium channel modification by arginine methylation have been documented. Available electrophysiological data are consistent with an increase in sodium current density, most likely due to enhanced Na_V_ membrane expression (Beltran-Alvarez et al. 2013; Baek et al. 2014). Additionally, the group of Trimmer reported considerable acceleration in Na_V_1.2 recovery from inactivation when arginine methylation was enhanced (Baek et al. 2014). Remarkably, arginine methylation is an example of PTM conservation among Na_V_ isoforms, even if catalysed by different enzymes: Na_V_1.2 is methylated by PRMT8 (mostly expressed in the CNS), while Na_V_1.5 methylation is catalysed by PRMT3 and -5 (ubiquitously expressed).

### Other known post-translational modifications

We would like to mention that sodium channels have long been known to undergo cysteine modifications including *S*-palmitoylation (Schmidt and Catterall 1987; Bosmans et al. 2011), and S-nitrosylation (Renganathan et al. 2002). Methionine oxidation of sodium channels has previously been reviewed (Cui et al. 2012). SUMOylation of the Na_V_1.7 isoform has been described, but available data suggest that SUMOylation may not be conserved in CNS Na_V_ isoforms (Dustrude et al. 2013).

Another well-known PTM, *N*-glycosylation, has been mostly studied in the cardiac isoform of the sodium channel, and several excellent reviews have recently been published (Baycin-Hizal et al. 2014; Marionneau and Abriel 2015). Perhaps the latest studies are those from the Chatelier and the Decosterd–Abriel groups, which have proposed alternative trafficking pathways for differentially glycosylated Na_V_, using Na_V_1.5 and Na_V_1.7 as study models (Mercier et al. 2015; Laedermann et al. 2013, respectively).

### Other possible post-translational modifications?

The advent of large-scale proteomics including the publication of human proteome maps is revolutionising life sciences. The ion channel field can also benefit from the analysis of big data to anticipate and identify challenges and opportunities, particularly in the field of PTMs. With this in mind, we searched Phosphositeplus (Hornbeck et al. 2015) for PTMs of Na_V_ isoforms. The database contains potentially novel sodium channel modifications including Lys acetylation, which is reported for Na_V_1.1, Na_V_1.2, Na_V_1.3, Na_V_1.5 and Na_V_1.6, and Lys methylation, which is included for Na_V_1.2 and Na_V_1.6.

Although promising at first sight, available data must be regarded with care. Conservation of the reported post-translationally acetylated or methylated Lys site among Na_V_ isoforms was very low. The finding worth mentioning was interspecies conservation of Na_V_1.1 acetylation at K1948 in human and mouse samples. Although K1948 acetylation was observed in unrelated experiments, it must be noted that the source of tissue was not brain but colon cancer.

### Cross-talk between sodium channel PTMs

Cross-talk, or interplay, between PTMs includes the regulatory mechanisms by which PTMs work together to determine protein function. Cross-talk between sodium channel phosphorylation, and arginine methylation, has been reported. The group of Trimmer reported cross-talk between Na_V_1.2 arginine methylation and phosphorylation (Baek et al. 2014). In this study, the authors studied Na_V_1.2 PTMs in the rat brain. Na_V_1.2 was immunopurified, digested and subjected to mass spectrometry analysis. An initial observation was that detected Na_V_1.2 peptides harboured either arginine methylation or phosphorylation, but not both PTMs on the same peptide. Convincingly, these two PTMs were reciprocally regulated in response to acute seizure: e.g., R563 methylation (see also Table 2) increased but S554 and S568 phosphorylation decreased after induction of seizure in rats (Baek et al. 2014). The most likely mechanism for this interplay between sodium channel arginine methylation and phosphorylation is the modification of kinetic specificity constants of serine phosphorylation upon methylation of a neighbouring arginine, and viceversa (Beltran-Alvarez et al. 2015). Nevertheless, the functional consequences of phosphorylation—arginine methylation cross-talk remain to be elucidated.

Additionally, cross-regulation between Na_V_1.6 phosphorylation, and ubiquitylation, has been observed. On the one hand, Na_V_1.6 is phosphorylated by p38 MAPK at position S553. On the other, Na_V_1.6 is ubiquitylated by Nedd4-2 after recognition of the PY motif (Pro-Ser-Tyr) at the *C* terminus of the channel. Results from the group of Dib-Hajj suggested that S553 phosphorylation enables further Na_V_1.6 ubiquitylation and internalisation of the channel (Gasser et al. 2010). A similar mechanism has recently been proposed for Na_V_1.2 whereby phosphorylation of T1966 by GSK3 primes recognition by Nedd4-2 via the Na_V_1.2 PY motif (PPSY_1975_), (James et al. 2015).

### Regulation of brain sodium channels by proteases

Voltage-gated sodium channel density has long been known to be regulated by proteases under normal (Paillart et al. 1996) and stress conditions (Iwata et al. 2004). Among the most important proteases in mammalian cells stand the calpains, which target hundreds of proteins (Grimm et al. 2012). The group of Meany has revealed the bases of calpain-dependent proteolysis of Na_V_1.2.

Using rat brain homogenates, they showed that calpain cleaves Na_V_1.2 (but not Na_V_1.1) at two sites, i.e., the interdomain linkers between domains I and II, and between domains II and III (von Reyn et al. 2009). Intriguingly, most of the calpain sodium channel fragment products localise at the plasma membrane 6 h after calpain activation, and possibly interact (von Reyn et al. 2009). Perhaps the simplest explanation is that distinct sodium fragments still retain the protein–protein interactions that hold the sodium channel macromolecular complex together, and thus control the break-down of the complex. A more thought-provoking alternative is that sodium channel post-translational proteolysis creates new proteins with modified biological activities.

The group of Meany has dissected the mechanisms of Na_V_1.2 proteolysis in cellular and mouse models of neuronal injury (von Reyn et al. 2012; Schoch et al. 2013), opening opportunities for treatment and therapy of traumatic brain injury. In this line, other researchers have recently described the beneficial effect of calpain inhibitors on brain sodium channel expression and electrophysiology in a model of diabetic neuropathy (Kharatmal et al. 2015).

The other example of sodium channel processing by proteases is the excision of the initiation methionine by aminopeptidases. This has been shown for Na_V_1.5 (followed by *N*-terminal acetylation of the resulting initiation alanine) in cardiac disease (Beltran-Alvarez et al. 2014). Whether Na_V_1.5 or other Na_V_ isoforms are devoid of Met residues (or post-translationally acetylated) in normal tissue is unknown.

## Conclusions and perspective

Research in the voltage-gated sodium channel field has grown linearly for the last 20 years. While the interest in transcriptional mechanisms regulating sodium channel expression has also grown steadily, we have observed an exponential trend in the number of publications related to sodium channel post-translational regulation. We predict that this growth will keep pace over the coming years. The aim of this review was to provide the current state of the art of the transcriptional and post-translational regulation of sodium channels, and thus set the ground for further research opportunities and discoveries.

Our understanding of transcriptional mechanisms governing brain sodium channel expression is far from comprehensive, and the ongoing research efforts of the ENCODE Consortium will surely encourage groups around the globe to dissect the molecular mechanism controlling Na_V_ transcription. Analogously, there are new questions in the field of PTM of sodium channels, in particular related to cross-talk among co-occurring types of PTM. As an example, the functional consequences of the interplay between phosphorylation and arginine methylation are intriguing, because the latter is thought to be a rather stable PTM (Bedford and Clarke 2009). The dynamic sequence of PTM events, thus, acquires vital relevance. Our incomplete understanding of proteolysis and degradation pathways of sodium channels also warrants further research in the area.

From the point of view of cell biology, biochemistry and electrophysiology, we predict that major advances in our understanding of Na_V_ regulation will be made in two main directions. First, systems biology approaches will integrate knowledge on Na_V_ biology, including transcriptional and post-translational regulation. This may be done using mathematical models and simulations of protein expression, function and degradation at the single molecule level, or, e.g., at the level of action potentials. Second, structural insights into whole sodium channel proteins, or isolated domains, will provide the framework to rationalise possible interactions between PTMs.

Additionally, research on Na_V_ is intrinsically associated to biomedical sciences, given the prominent relevance of these channels in a range of neurological and cardiac disorders. In this respect, in the following years we expect reports on quantitative experiments identifying changes in PTM patterns in disease (some recent examples include Baek et al. 2014; and Herren et al. 2015). The effect of sodium channel proteolysis in major neurological diseases is also an emerging field of research (Corbett et al. 2013), which includes the identification of genetic mutations in proteases affecting sodium channel levels (Kim et al. 2014).
